# Supplementary material for: Aerosol size determination via light scattering of viruses and protein complexes
Source: Commun Phys. 2025 Apr 12;8(1):155. doi: 10.1038/s42005-025-02076-3 (PMC11993359; doi:10.1038/s42005-025-02076-3)
Supplement: Supplementary file 2 — Supplementary Information [file 42005_2025_2076_MOESM2_ESM.pdf]

Supplementary Information

Aerosol size determination via light scattering of viruses and protein complexes

Lena Worbs, Tej Varma Yenupuri, Tong You, Filipe R.N.C. Maia

March 27, 2025

Supplementary Note 1

Sample Details

We used NIST-traceable monodisperse polystyrene spheres (PS) (Fischer Scientific) with diameters between 18 and 59 nm suspended in an aqueous 20 mM ammonium acetate solution of $10^{12} - 10^{13}$ particles/ml and biosamples (ferritin, ribosomes and bacteriophage MS2) for the measurements. Apo-ferritin (from Equine Spleen) was purchased from Sigma-Aldrich (CAS number 9013-31-4). The particles were put in 20 mM AmAc to the desired particle concentration. Ribosomes were prepared in-house according to the protocol given in [1]. The MS2 sample was prepared following a modified protocol based on ([2]). The virus was propagated using E. coli strain K12 (ATCC 10798). After the precipitation steps and re-suspension in Tris buffer, the sample was further purified on a Sepharose CL-4B (Cytiva) column (500 mL). Peak fractions were pooled and precipitated overnight at 4°C using 10% PEG 6000 and 0.5 M NaCl (from a 5 M NaCl solution). The precipitate was then centrifuged at 27000xg for 30 min, and the resulting pellet was re-suspended in Tris buffer. Prior to injection, the Tris buffer was exchanged to 20 mM AmAc using aPD Minitrap G-25 column (Cytiva).

For particle sizing, we used a differential mobility analyser (TSI model 3080) and a condensation particle counter (TSI model 3786) (DMA-CPC). The peak size from the manufacturer, the measured peak size in the DMA and the sample concentration is shown in Supplementary Table 1. We measured a different peak size for the PS sample than the manufacturers' given size using the DMA-CPC setup. Especially the 50 nm PS size is off. A dynamic light scattering measurement of the 50 nm PS sample returned a diameter of 58.5 nm. We decided to use the peak sizes from the DMA-CPC measurements for calibration instead of the manufacturers' provided sizes.

Sample name	Mean size (nm)	DMA peak size (nm)	range limits ($1/e^2$) (nm)	concentration in solution (particles/ml)
ferritin	12 [3]	13.6	—	6.3E+14
ribosome 70S	21 [4]	19.5	—	6E+14
bacteriophage MS2	27 [5]	25.9	—	6E+12
20 nm PS	23 ± 2	18.8	12 - 34	8.7E+13
30 nm PS	31 ± 3	28.9	13 - 42	2.6E+13
40 nm PS	41 ± 4	42.9	21 - 53	1.1E+13
50 nm PS	51 ± 3	59.4	46 - 69	5.6E+12

Supplementary Table 1: Sample details.

The particle size histogram is measured with the DMA-CPC assembly and the distributions are shown in Supplementary Figure 1. The distributions were measured with the same sample concentration, liquid and gas flow rate and capillary as for the scattering measurements.

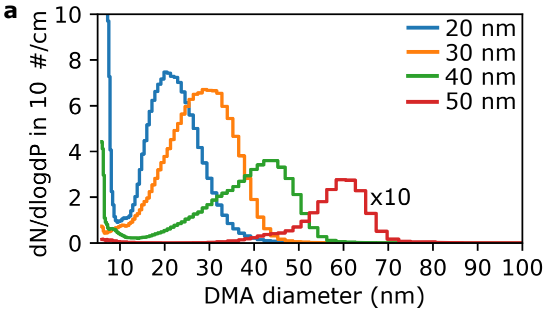

Supplementary Figure 1: DMA-CPC size distributions for the used samples. a.) PS size histograms and b.) the bioparticles' size histograms measured with a DMA-CPC. The 50 nm PS trace is multiplied by 10 to be visible.

Supplementary Note 2

MS2 Buffer Runs

We assume that the small particle sizes in the MS2 measurements correspond to the sample buffer forming particles from non-evaporative buffer. To confirm this hypothesis, we first had to calibrate our setup again using PS spheres due to problems with the laser used for the experiments presented in the main manuscript. We used a pulsed Nd:YAG laser (Quantel Brio, $\lambda = 532$ nm, 15 Hz rep. rate, 10 ns pulse width, 75 mJ pulse energy). The calibration curve that we obtained in the same way as described in the main manuscript is shown in Figure S2a. The linear slope is extracted to be 0.192 with a standard deviation of 0.006.

We prepared MS2 buffer solution without virus particles inside and electrosprayed the solution. The MS2 buffer solution is a Tris buffer. The DMA-CPC size histogram is shown in Figure S2b as the orange curve. Only one peak with a peak diameter of $<$ 15 nm is visible. Then, we connected the ESI source to the expermiental setup to measure the scattering from the generated particles. We collected 3000 frames with 74 focused particle hits analysed. The size distribution of the measured particles is shown as the blue size histogram in Figure S2b. Only the falloff from small particle sizes towards larger particles is visible. Overall, the particle sizes from the scattering appear a bit larger than the particle sizes measured with the DMA-CPC setup. This may be caused by a slightly larger flow rate in the ESI source. We do not observe a second peak in the size histogram as presented in the main manuscript.

In addition, we also injected 20 mM AmAc solution. In this case, we did not record a single particle hit in our scattering data.

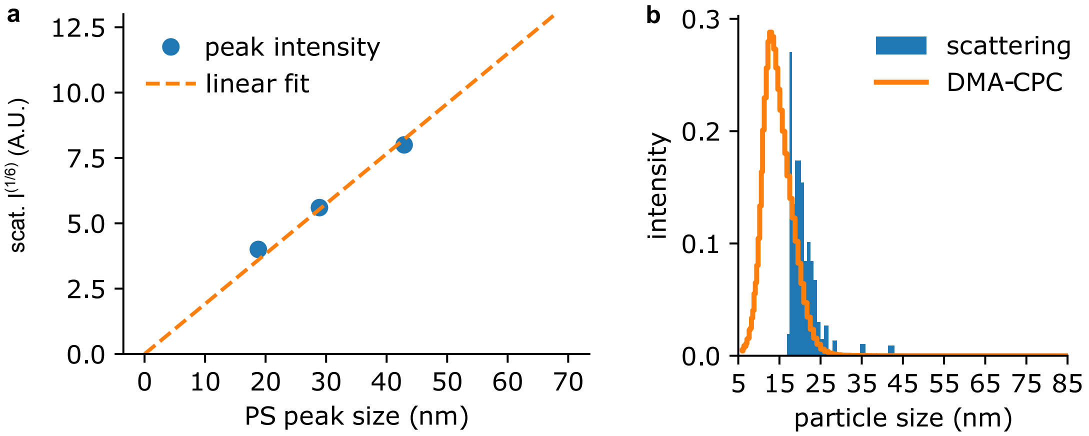

Supplementary Figure 2: MS2 Buffer measurement results. a.) Calibration curve using the brio laser. b.) MS2 buffer measurement. Blue: size histogram from the scattering experiment and orange: size histogram from the DMA-CPC measurement.

Supplementary Figures

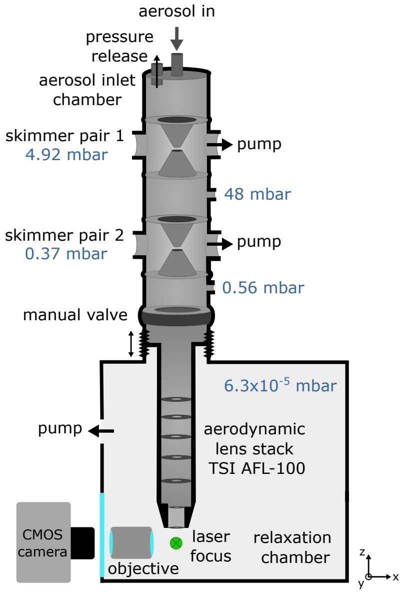

Supplementary Figure 3: Schematic setup. The generated aerosol enters two skimmer pairs for excess gas removal before entering the aerodynamic lens for particle-beam generation. In the interaction chamber, the particle beam is intersected by a focused laser beam. The scattering by the particles is recorded with a camera-based microscope system.

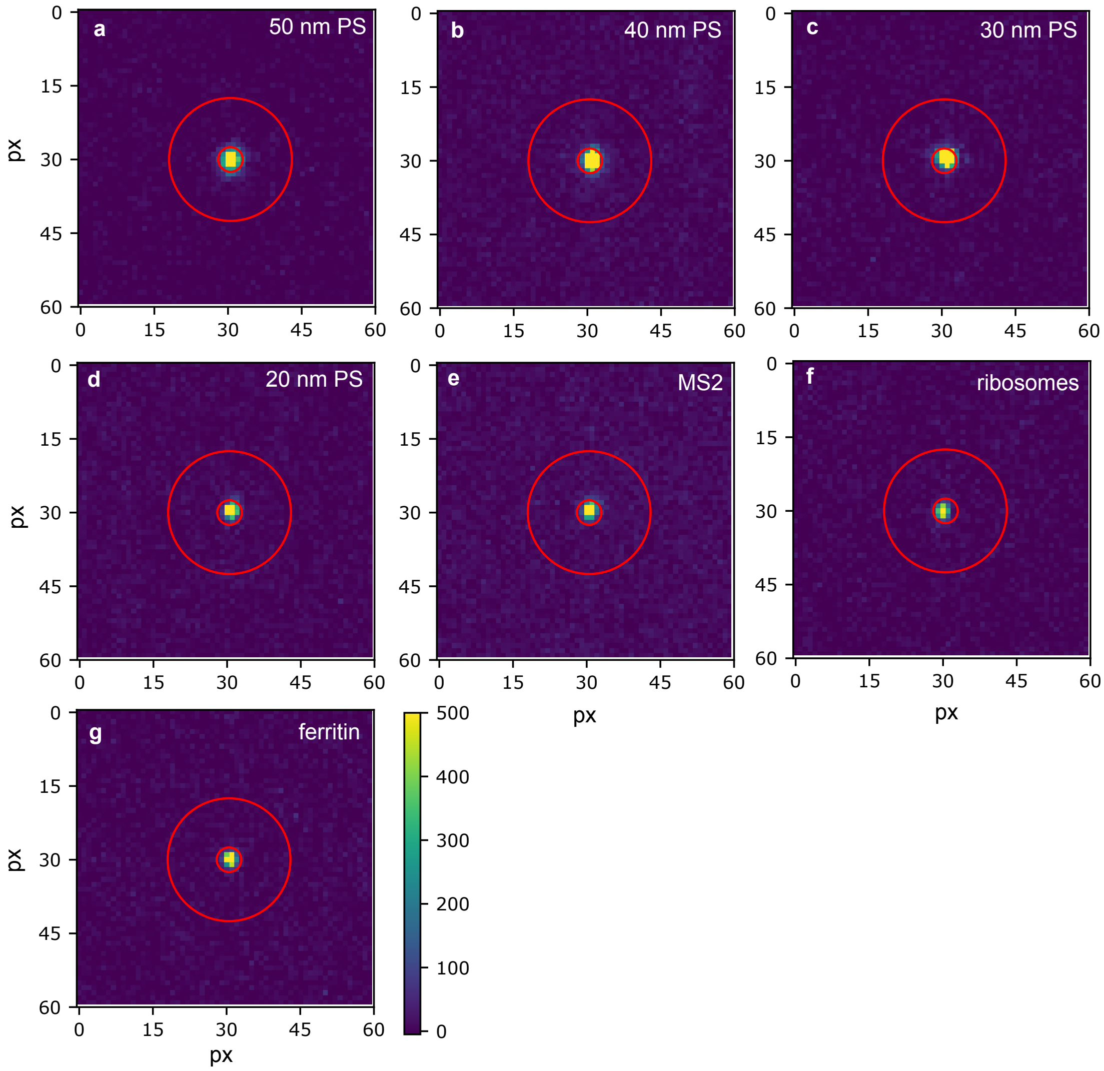

Supplementary Figure 4: Example particle hits for the different particle size and species. The two red circles represent the 5 and 25 px circular windows used for analysis. All hits shown here fall into the focused particle hit category and are used in further data analysis.

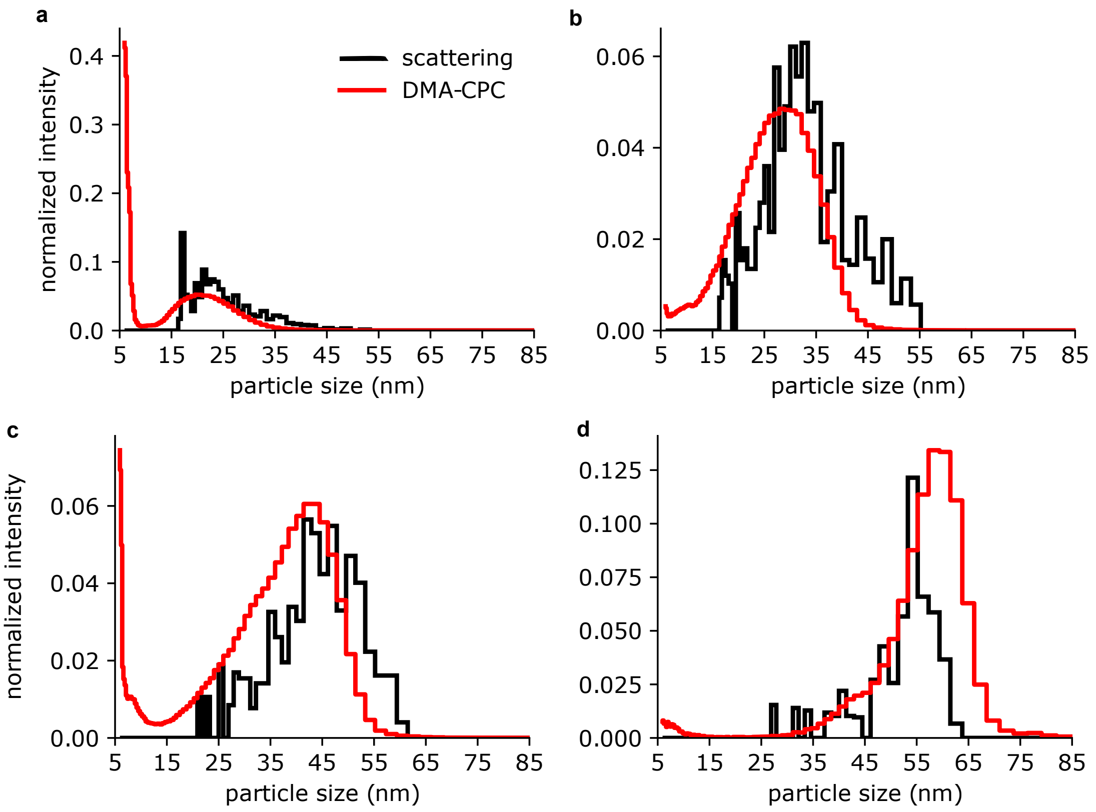

Supplementary Figure 5: Polystyrene DMA and laser scattering comparisons. Retrieved PS size histograms from the scattering data (black) compared to the measured size distribution using the DMA-CPC (red) for the used PS samples. a.) 20 nm PS, b.) 30 nm PS, c.) 40 nm PS and d.) 50 nm PS.

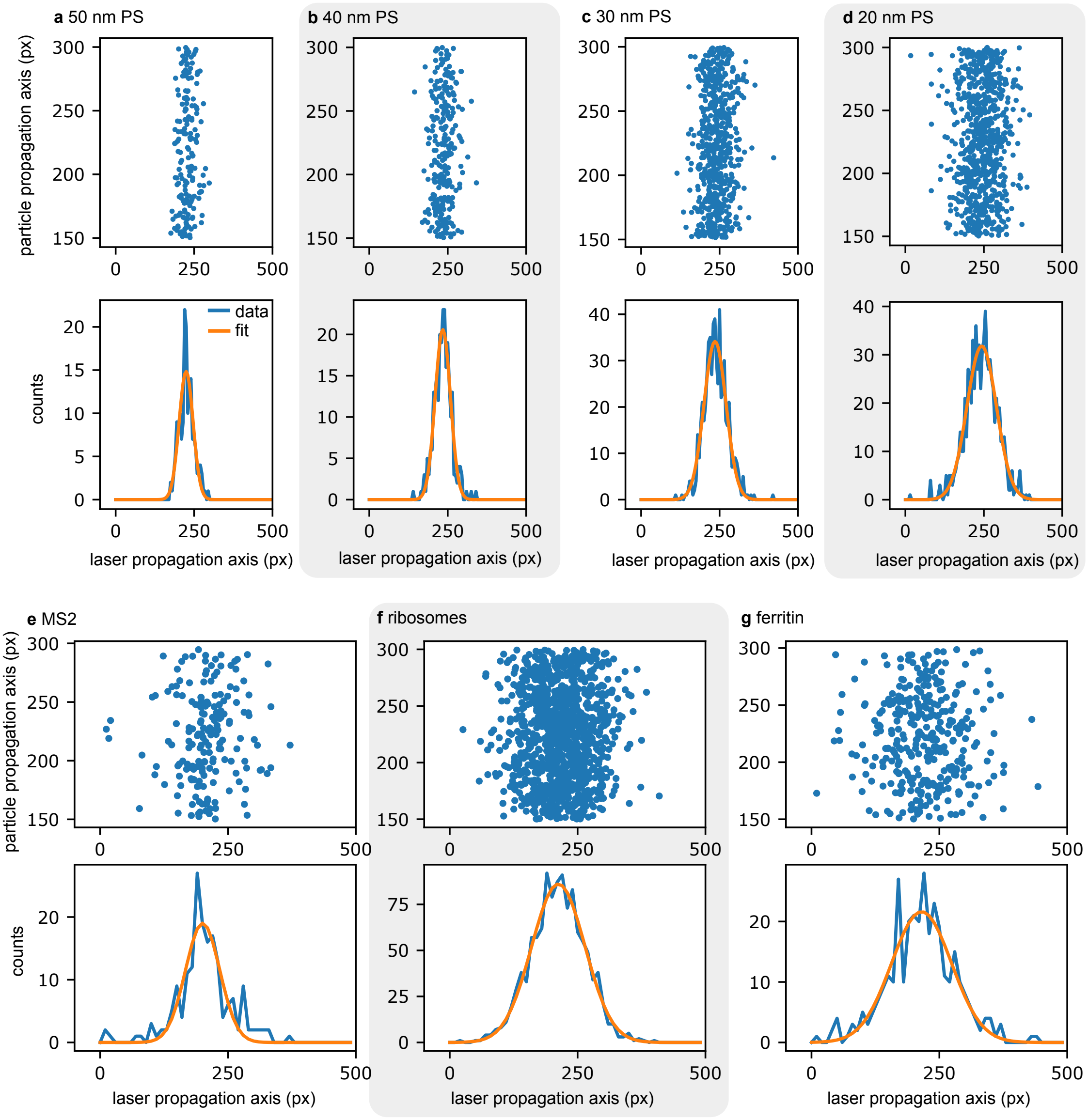

Supplementary Figure 6: Particle positions and particle beam profile. The upper row shows the scatter plot of the particles' position and the lower row shows the projection of the particle positions onto the laser propagation axis. The projection of the particle positions onto the laser propagation axis reveals the 1D particle beam profile and a Gaussian fit is used to determine the full width at half maximum (FWHM). The particle beam width we retrieve is 70, 53, 36, 33, 88, 78, and 50 μm for the 20, 30, 40, 50 nm PS, ferritin, ribosome and MS2 sample, respectively. All measurements were taken at a distance of 2.0 mm from the aerosol injector exit.

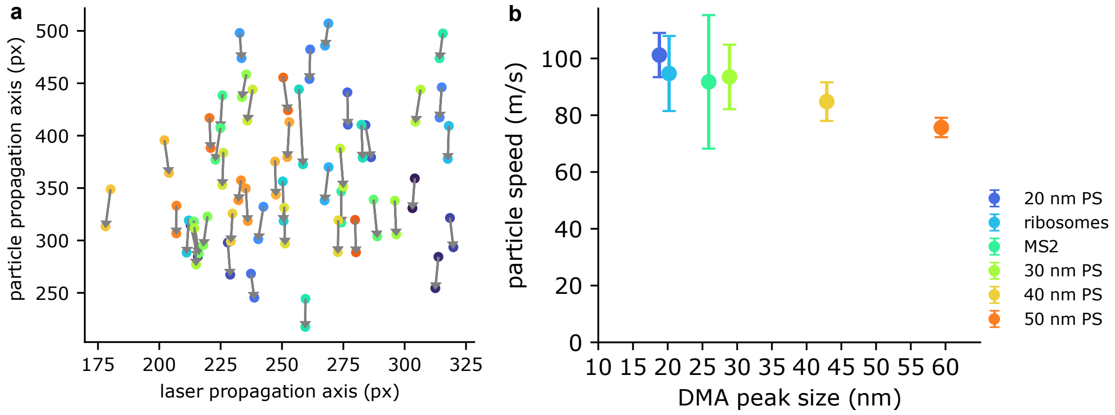

Supplementary Figure 7: Particle speed measurements. a.) Particle positions of found double hits while injecting MS2 particles. The particle position difference and the time delay between the two pulses is used to determine the speed of the particles. b.) Mean speed of the particles depending on the particle size. The error is given as the standard deviation of the speed distribution. For ferritin, no speed could be determined.

Supplementary References

[1] Johansson, M., Bouakaz, E., Lovmar, M. & Ehrenberg, M. The kinetics of ribosomal peptidyl transfer revisited. *Molecular Cell* **30**, 589–598 (2008). URL https://www.sciencedirect.com/science/article/pii/S1097276508002931.

[2] Mall, A. *et al.* Observation of aerosolization-induced morphological changes in viral capsids (2024). URL https://arxiv.org/abs/2407.11687. 2407.11687.

[3] Dostalova, S. *et al.* Apoferritin as an ubiquitous nanocarrier with excellent shelf life. *Int. J. Nanomedicine* **12**, 2265–2278 (2017).

[4] Schuwirth, B. S. *et al.* Structures of the bacterial ribosome at 3.5 Å resolution. *Science* **310**, 827–834 (2005). URL https://www.science.org/doi/abs/10.1126/science.1117230.

[5] Kuzmanovic, D. A., Elashvili, I., Wick, C., O'Connell, C. & Krueger, S. Bacteriophage ms2: Molecular weight and spatial distribution of the protein and rna components by small-angle neutron scattering and virus counting. *Structure* **11**, 1339–1348 (2003). URL https://www.sciencedirect.com/science/article/pii/S0969212603002223.
